# IL6 receptor inhibitors: exploring the therapeutic potential across multiple diseases through drug target Mendelian randomization

**DOI:** 10.3389/fimmu.2024.1452849

**Published:** 2024-08-20

**Authors:** Chong Fu, Longquan Wang, Wenjiao Cai

**Affiliations:** ^1^ Department of Gastroenterology, Anqing Municipal Hospital, Anqing, Anhui, China; ^2^ Department of Geriatric Medicine, The First Affiliated Hospital of Wannan Medical College, Wuhu, Anhui, China; ^3^ Department of Nephrology, Anqing Municipal Hospital, Anqing, Anhui, China

**Keywords:** IL-6 receptor, Mendelian randomization, idiopathic pulmonary fibrosis, Parkinson’s disease, type 2 diabetes

## Abstract

**Background:**

High interleukin-6 levels correlate with diseases like cancer, autoimmune disorders, and infections. IL-6 receptor inhibitors (IL-6Ri), used for rheumatoid arthritis and COVID-19, may have wider uses. We apply drug-target Mendelian Randomization (MR) to study IL-6Ri’s effects.

**Method:**

To simulate the effects of genetically blocking the IL-6R, we selected single nucleotide polymorphisms (SNPs) within or near the IL6R gene that show significant genome-wide associations with C-reactive protein. Using rheumatoid arthritis and COVID-19 as positive controls, our primary research outcomes included the risk of asthma, asthmatic pneumonia, cor pulmonale, non-small cell lung cancer, small cell lung cancer, Parkinson’s disease, Alzheimer’s disease, ulcerative colitis, Crohn’s disease, systemic lupus erythematosus, type 1 diabetes, and type 2 diabetes. The Inverse Variance Weighted (IVW) method served as our principal analytical approach, with the hypotheses of MR being evaluated through sensitivity and colocalization analyses. Additionally, we conducted Bayesian Mendelian Randomization analyses to minimize confounding and reverse causation biases to the greatest extent possible.

**Results:**

IL-6 inhibitors significantly reduced the risk of idiopathic pulmonary fibrosis (OR= 0.278, 95% [CI], 0.138–0.558; P <0.001), Parkinson’s disease (OR = 0.354, 95% CI, 0.215–0.582; P <0.001), and positively influenced the causal relationship with Type 2 diabetes (OR = 0.759, 95% CI, 0.637–0.905; P = 0.002). However, these inhibitors increased the risk for asthma (OR = 1.327, 95% CI, 1.118–1.576; P = 0.001) and asthmatic pneumonia (OR = 1.823, 95% CI, 1.246–2.666; P = 0.002). The causal effect estimates obtained via the BWMR method are consistent with those based on the IVW approach. Similarly, sIL-6R also exerts a significant influence on these diseases.Diseases such as Alzheimer’s disease, Crohn’s disease, pulmonary heart disease, systemic lupus erythematosus, Type 1 diabetes, Non-small cell lung cancer and ulcerative colitis showed non-significant associations (p > 0.05) and were excluded from further analysis. Similarly, Small cell lung cancer were excluded due to inconsistent results. Notably, the colocalization evidence for asthmatic pneumonia (coloc.abf-PPH4 = 0.811) robustly supports its association with CRP. The colocalization evidence for Parkinson’s disease (coloc.abf-PPH4 = 0.725) moderately supports its association with CRP.

**Conclusion:**

IL-6Ri may represent a promising therapeutic avenue for idiopathic pulmonary fibrosis, Parkinson’s disease, and Type 2 diabetes.

## Introduction

Excessive production of IL-6 is characteristic of many rheumatic diseases, including Rheumatoid Arthritis, Juvenile Idiopathic Arthritis, and Adult-Onset Still’s Disease (1). IL-6Ri are increasingly used when traditional treatments with DMARDs, corticosteroids, and non-steroidal anti-inflammatory drugs prove ineffective, particularly in conditions such as RA, systemic Juvenile Idiopathic Arthritis, and Castleman’s disease (2). Recent advances in understanding the pathogenesis of rheumatic diseases have expanded the use of IL-6Ri to other rheumatic conditions, including AOSD, Giant Cell Arteritis, Behçet’s Disease, and Polymyalgia Rheumatica, thus positioning IL-6 or its receptor blockade as a novel strategy for managing certain rheumatic ailments. IL-6 inhibitors include Tocilizumab, Sarilumab, Sirukumab, Clazakizumab, Olokizumab, MEDI-5117, and ALX-0061 (3). Currently, only Tocilizumab and Sarilumab are commercially available, with Tocilizumab being the most widely used internationally, while other inhibitors are either in development or under research (4).

IL-6 is produced by a variety of cells, including hepatocytes, T cells, B cells, fibroblasts, monocytes, mesangial cells, keratinocytes, endothelial cells, and many tumor cells (5). The IL-6R is an 80 kDa type I transmembrane protein primarily expressed on immune effector cells such as T cells, B cells, monocytes, neutrophils, and macrophages, as well as some non-immune effector cells, including pancreatic and hepatic cells. IL-6R exists in two forms: a membrane-bound version (mIL-6R) and a soluble version (sIL-6R) (6). The binding of IL-6 to mIL-6R can induce homodimerization of gp130, forming a high-affinity IL-6/IL-6R/gp130 complex. The sIL-6Rs are produced either through the cleavage of mIL-6R by the protease ADAM-17 or via selective mRNA splicing. IL-6 can also bind to circulating sIL-6R, forming complexes with gp130 (7). The binding of IL-6 to mIL-6R activates the classical signaling pathway, while its interaction with sIL-6R initiates the trans-signaling pathway, both of which engage with membrane-bound glycoprotein 130 (mgp130) to trigger cascades involving the Janus kinase/signal transducer and activator of transcription (JAK/STAT) and the mitogen-activated protein kinase (MAPK) pathways (8). These complexes induce tyrosine phosphorylation in the cytoplasmic domain of gp130, which promotes the recruitment of STAT-3, leading to the expression of pro-inflammatory genes and suppressor of cytokine signaling proteins (SOCS) (9). The activation of RAS/mitogen-activated protein kinases (MAPKs) mediates the phosphorylation and activation of nuclear factor (NF)-IL-6, which binds to the IL-6 response elements in the promoter regions of acute-phase gene, inducing the production of acute-phase proteins. The IL-6/IL-6R/gp130 complex also activates JAK-1 and JAK-2 kinases, as well as downstream transcription factors such as STAT1, STAT3, and phosphoinositide 3-kinase (PI3K). The activated STATs translocate to the nucleus, regulating several genes, while the activated PI3K in turn activates the serine/threonine-protein kinase (B/AKT). The activation of JAK/STATS/PI3K and MAPK/ERK pathways mediated by IL-6 can induce a broad spectrum of immune responses (10).

MR is an instrumental variable analysis method that employs SNPs from genome-wide association studies (GWAS) as genetic tools to estimate the causal effects of exposures on outcomes. Compared to observational studies, MR’s advantage lies in leveraging the random allocation of alleles to circumvent biases from unobserved confounders, such as lifestyle factors and other environmental impacts, as well as issues related to reverse causality (11). This study utilizes drug-target Mendelian Randomization analysis, which employs genetic variants of simulated pharmacological inhibitions as instrumental variables. By conducting regression analysis, this method elucidates the long-term effects of medications and enhances causal inferences about these drug targets’ potential impact on diseases. Evidence suggests that drug-target MR effectively identifies targets with up to 70% efficiency (12). We have gathered recently published GWAS summary-level statistics to explore the causal relationships between genetically predicted IL6R inhibition and 15 diseases, including COVID-19, rheumatoid arthritis, asthma, asthmatic pneumonia, cor pulmonale, non-small cell lung cancer, small cell lung cancer, Parkinson’s, Alzheimer’s disease, ulcerative colitis, Crohn’s disease, systemic lupus erythematosus, and types 1 and 2 diabetes through drug-target MR analysis.

## Methods

### Study design and data resources

**Figure 1:**
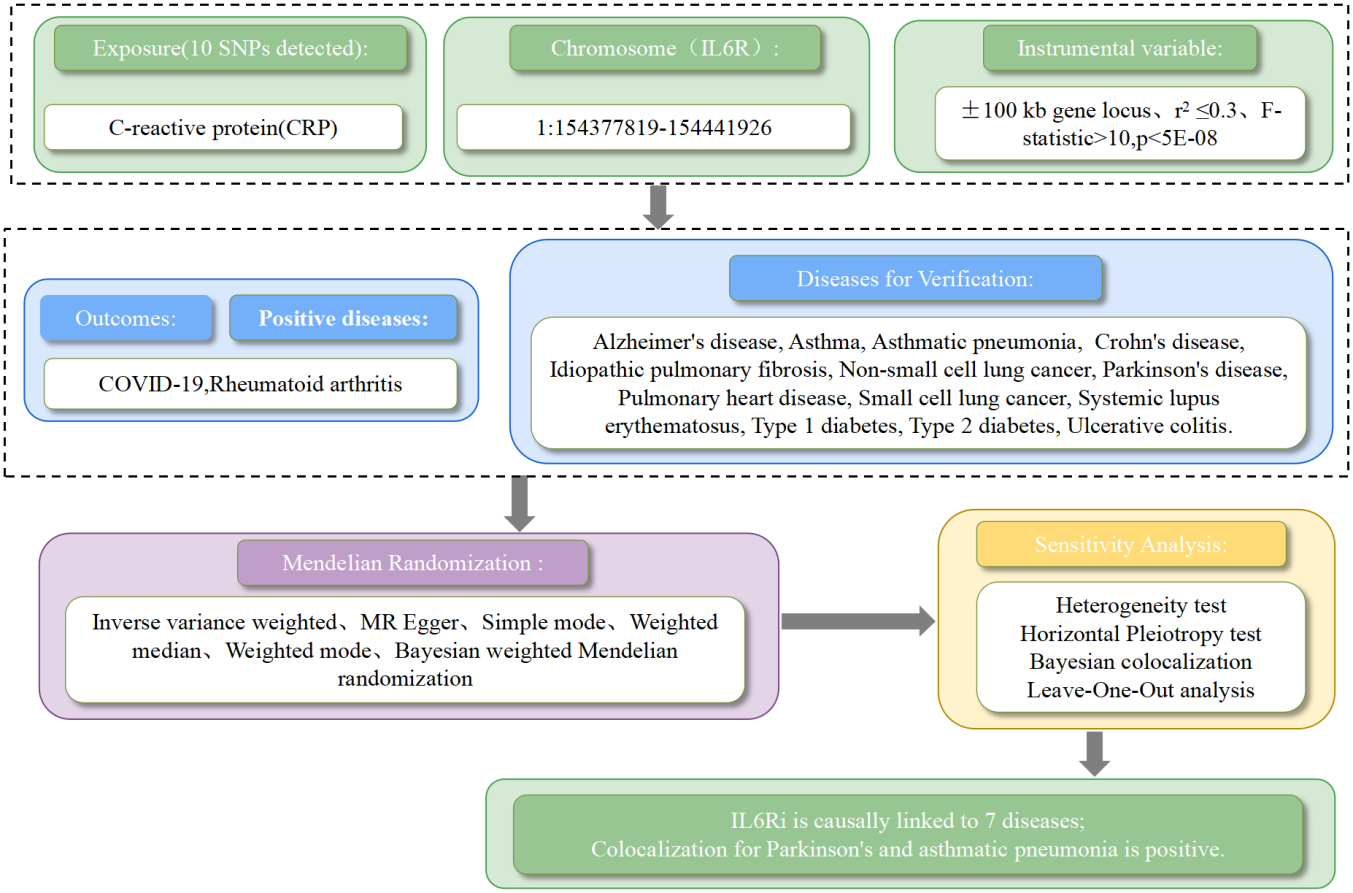
The workflow of drug target mendelian randomization study that exhibits causality between IL6 receptor inhibitors and multiple diseases.

**Figure 1** summarizes the design of this study. C-reactive protein (CRP) GWAS data was derived from a study encompassing 204,402 Europeans(GWAS ID:ieu-b-35). CRP was selected as a biomarker because it is well-documented that pharmacological inhibition of IL-6R can reduce CRP levels, a finding substantiated by clinical trials (13, 14). By acquiring instrumental variables that target IL6R to diminish CRP levels, we can simulate the effects of IL6Ri (15). These instrumental variables are SNPs located within ±100kb of the IL6R gene locus, which are associated with CRP levels and meet the genome-wide significance threshold set at p<5×10^-8. To mitigate the impact of strong linkage disequilibrium (LD) on the results, an LD threshold (r^2<0.3) was established. Ultimately, 10 significant IL6R SNPs were retained (**Supplementary Material 1**: **Supplementary Table S2**). To verify the absence of weak instrument bias in our selected instrumental variables, we employed the F-statistic, where an F > 10 indicates the absence of weak instrument bias, further validating the associative hypotheses. The formula for the F-statistic is F = [(N - K - 1)/K] × [R^2/(1 - R^2)], where N is the sample size of the exposure, K is the number of instrumental variables, and R^2 is the proportion of exposure variance explained by the instrumental variables.

We utilized fifteen diseases for our drug-target Mendelian Randomization analysis, with rheumatoid arthritis(GWAS ID:finn-b-RHEUMA_NOS,n=217134) and COVID-19(GWAS ID:ebi-a-GCST011077,n=1059456) serving as two positive control datasets, all sourced from European populations. Additionally, we compiled GWAS summary data sets for idiopathic pulmonary fibrosis (GWAS ID:finn-b-IPF,n=198014), asthma (GWAS ID:finn-b-J10_ASTHMA,n=156078),asthmatic pneumonia (GWAS ID:finn-b-ASTHMA_PNEUMONIA_AND_SEPSIS,n=140994), cor pulmonale (GWAS ID:finn-b-FG_PULMHEART,n=218792), non-small cell lung cancer (GWAS ID:finn-b-C3_LUNG_NONSMALL_EXALLC,n=175633), small cell lung cancer (GWAS ID:finn-b-C3_SCLC,n=218792),Parkinson’s disease(GWAS ID:finn-b-PDSTRICT,n=218473),Alzheimer’s disease (GWAS ID:finn-b-G6_ALZHEIMER,n=156078),ulcerative colitis (GWAS ID:finn-b-K11_ULCER,n=214620),Crohn’s disease (GWAS ID:finn-b-K11_CROHN,n=212356),systemic lupus erythematosus (GWAS ID:finn-b-M13_SLE,n=213683), type 1 diabetes (GWAS ID:finn-b-E4_DM1,n=189113), and type 2 diabetes (GWAS ID:finn-b-E4_DM2,n=215654)(**Supplementary Material 1**: **Supplementary Table S1**). These principal outcome datasets originated from the Finnish database, accessible via the MR-Base platform (https://www.mrbase.org/). There were no overlapping samples between the exposure and outcome GWAS, and all participants were of European descent.This research complies with the Strengthening the Reporting of Observational Studies in Epidemiology Using Mendelian Randomization(STROBE-MR) guidelines (16), and the corresponding checklist can be found in the Supporting Information (**Supplementary Material 1**: STROBE-MR checklist).

### MR analysis

IL-6Ri have been extensively utilized in the treatment of rheumatoid arthritis and COVID-19. Consequently, we employed GWAS summary data from these diseases as positive controls to validate the efficacy of our instrumental variables. Initially, we harmonized drug-target instrumental variables related to exposure with the outcome datasets. We implemented five MR methods to assess the causal association between these drug-target instrumental variables and the outcome datasets, including MR-Egger regression, Weighted Median Estimator (WME), Inverse Variance Weighted (IVW), Simple Mode, and Weighted Mode. The IVW method was chosen as the primary analytical approach because it provides the most precise estimates by analyzing each Wald ratio under the ideal condition that all instrumental variables are valid (17). Given that this represents an ideal scenario, it is crucial to corroborate these findings with other methods to ensure robustness and validity across various analytical conditions. Additionally, a Bayesian weighting strategy was used to simultaneously address measurement errors and pleiotropy, thus enhancing the robustness of our analysis (18).

### Sensitivity analyses

Heterogeneity tests were conducted using MR Egger and IVW methods. Cochran’s Q value was employed to evaluate the heterogeneity of genetic instruments, with p > 0.05 indicating no significant heterogeneity. MR Egger regression and MR PRESSO were used to assess pleiotropy of the genetic instruments, with p > 0.05 indicating no pleiotropy (19, 20). These steps help ensure the reliability of our instrumental variables in assessing causal relationships.

Bayesian colocalization analysis is employed to assess the probability that two traits share the same causal variant, facilitated by the ‘coloc’ package using default parameters (https://github.com/chr1swallace/coloc) (21). Bayesian colocalization offers posterior probabilities for five hypotheses, determining whether two traits share a single variant. This analysis evaluates support for five exclusive hypotheses: 1) associated with neither phenotype; 2) associated only with phenotype 1; 3) associated only with phenotype 2; 4) both phenotypes are associated, but with different causal variants; and 5) both phenotypes are associated with the same causal variant. Strong colocalization support is considered when the posterior probability of a shared causal variant (PH4) exceeds 0.8. Moderate colocalization is defined as a PH4 value between 0.5 and 0.8.

### Statistical power calculation

The statistical efficacy of the research was assessed using an online power calculator developed by Burgess (https://sb452.shinyapps.io/power/) (22). This comprehensive evaluation incorporated the total sample size, a significance level of 0.05, the variance explained by instrumental variables concerning exposure, and the ratio of exposure between the case and control groups, as well as the OR from the MR analysis. We established an objective to achieve a statistical power threshold of at least 0.8 to ensure that when the p-value is less than 0.05, the findings achieve statistical significance, thereby validating the scientific rigor of the results.

## Results

### Positive control analysis

**Figure 2:**
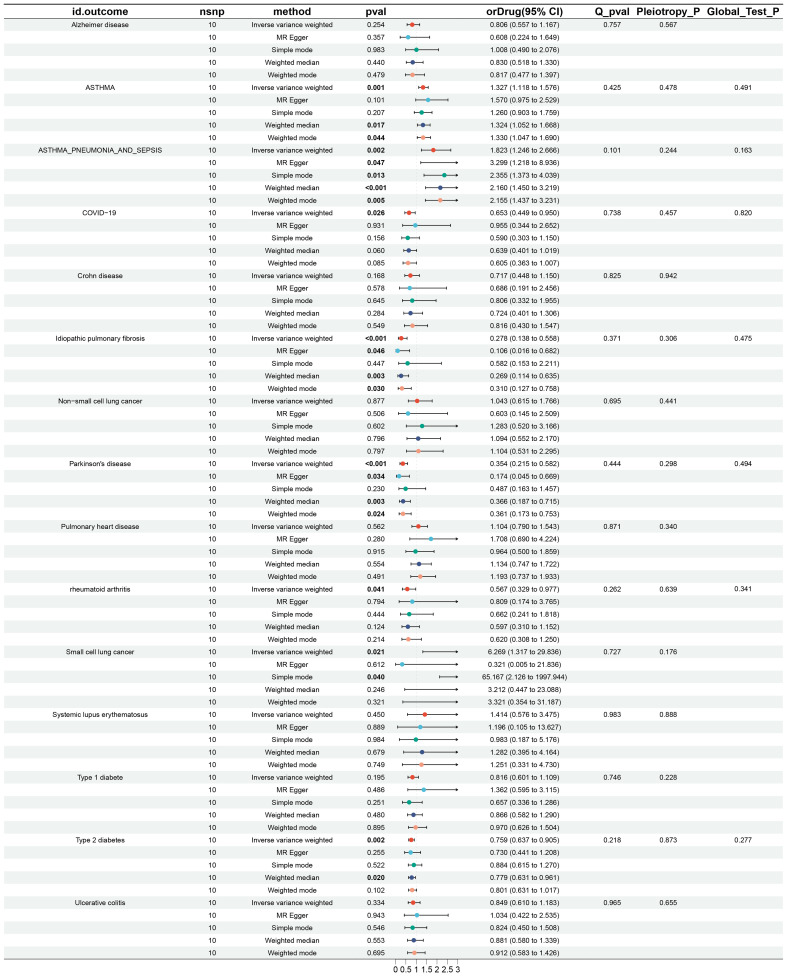
Results and forest plot of MR analysis of IL-6Ri’s causal relationship with 15 diseases.nsnp: The final number of SNPs used in the analysis. orDrug: The estimated effect of IL6Ri on diseases. Q_pval: The P value of the Cochran Q test. Pleiotropy_P: The P value of the MR-Egger regression intercept hypothesis test. Global_Test_P: The P value of the MR-PRESSO global test.

The causal effects of IL6Ri on fifteen diseases were analyzed using Mendelian Randomization (MR), setting a strict p-value threshold of 5 × 10^−8. Through rigorous criteria (r^2 < 0.3, kb = 100 KB), ten significant IL6Ri SNPs were identified. The F-statistics for these instrumental variables were all above 10, indicating the absence of weak instrumental bias and confirming the reliability of the results (**Supplementary Material 1**: **Supplementary Table S2**). Genetic variations in the IL6R target associated with inhibitors were linked to a reduced risk of rheumatoid arthritis and COVID-19. Specifically, there was a negative causal relationship between IL6Ri and rheumatoid arthritis (OR = 0.567, 95% CI, 0.329–0.977; P< 0.041) and COVID-19 (OR = 0.653, 95% CI, 0.449–0.950; P= 0.026). The causal effect estimates from the BWMR method were consistent with those based on the IVW method (**Figure 2**, **Supplementary Material 1**: **Supplementary Tables S3**, **S4**).

### The causal relationship between gene-simulated IL6Ri and multiple diseases

**Figure 3:**
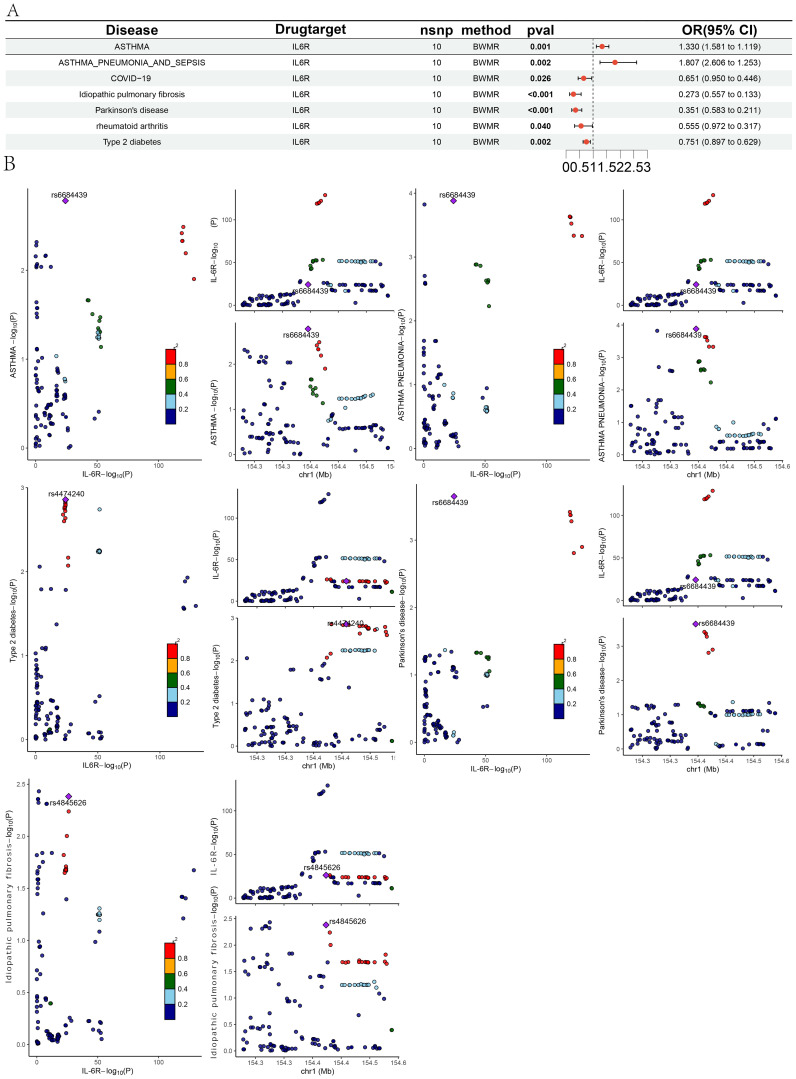
**(A)** Forest plot of BWMR analysis of IL-6Ri’s causal relationship with 15 diseases; **(B)** The results of colocalization analysis.

**Figure 4:**
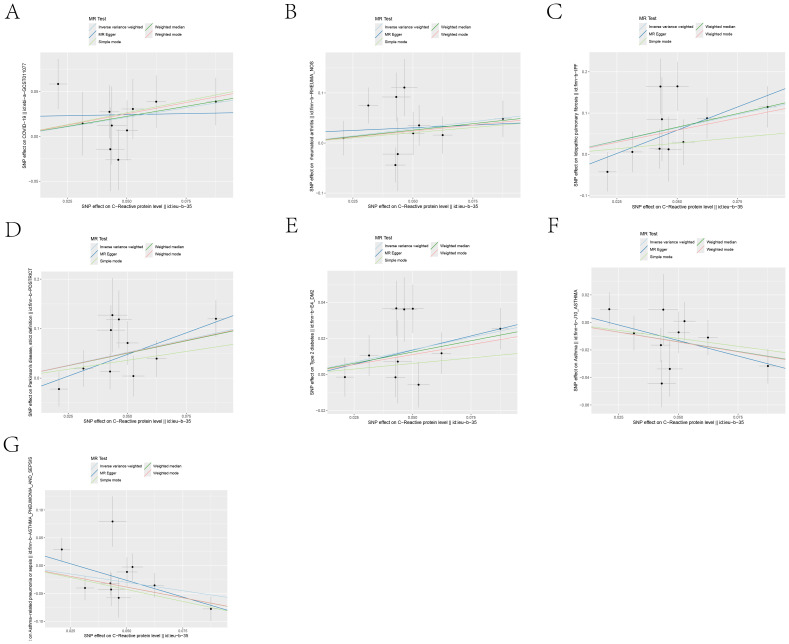
Scatter plots of the 5 MR analysis methods. The causal effect of IL-6R inhibitors on seven types of diseases, including: **(A)** COVID-19, **(B)** Rheumatoid arthritis, **(C)** Idiopathic pulmonary fibrosis, **(D)** Parkinson’s disease, **(E)** Type 2 diabetes, **(F)** Asthma, **(G)** Asthmatic pneumonia.The vertical axis denotes the influence of single nucleotide polymorphisms on exposure variables,the horizontal axis delineates their impact on outcome variables.

**Figure 5:**
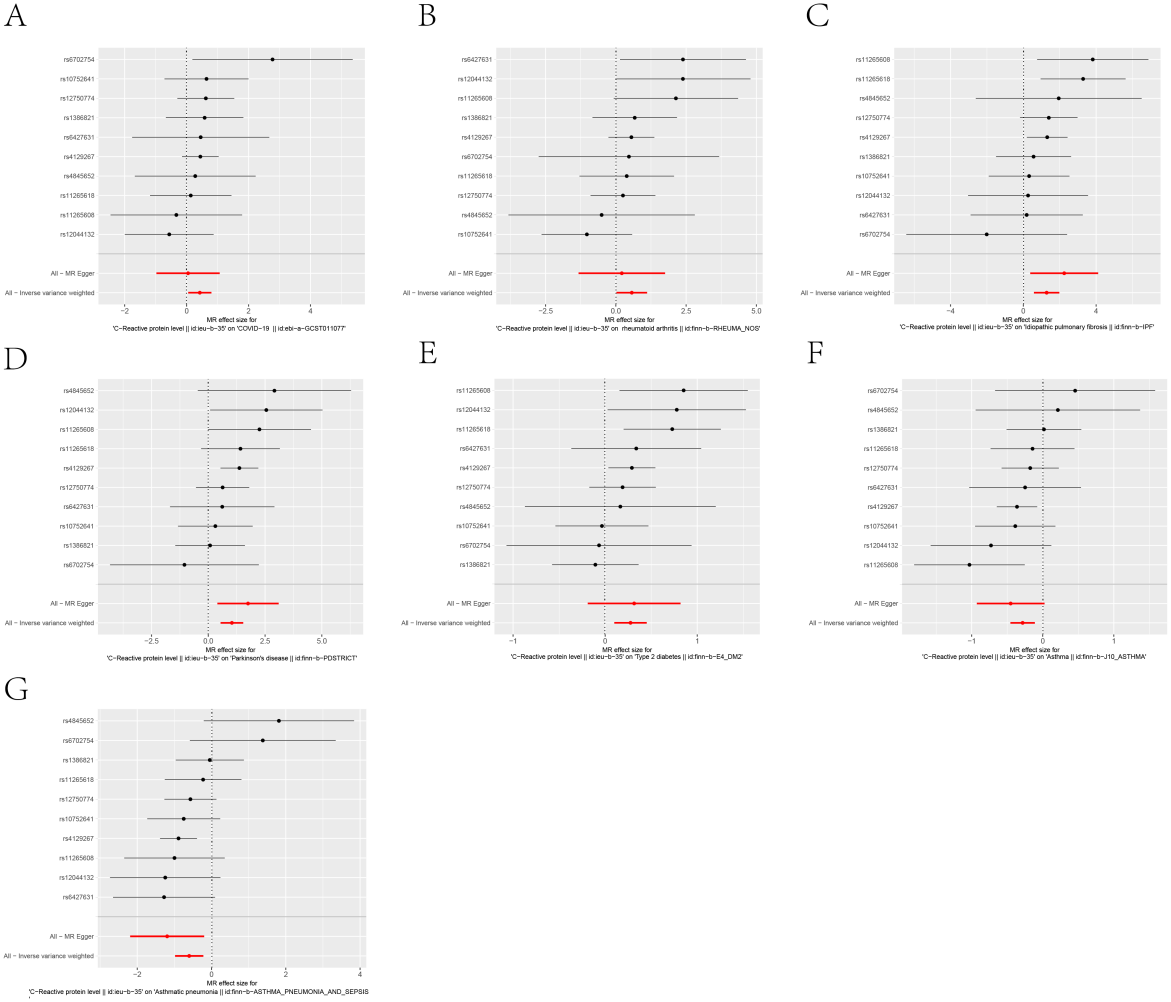
Forest plot of each SNP’s causal relationship with seven diseases, including: **(A)** COVID-19, **(B)** Rheumatoid arthritis, **(C)** Idiopathic pulmonary fibrosis, **(D)** Parkinson’s disease, **(E)** Type 2 diabetes, **(F)** Asthma, **(G)** Asthmatic pneumonia.The black dots represent the mean values,All-MR Egger and All-Inverse variance weighted represent the overall effect of all instrumental variables.

MR analysis revealed associations between IL6Ri and diseases such as idiopathic pulmonary fibrosis, Parkinson’s disease, type 2 diabetes, asthma, and asthmatic pneumonia. Specifically, there was a negative causal relationship between IL6Ri and idiopathic pulmonary fibrosis (OR = 0.278, 95% CI, 0.138–0.558; P <0.001), Parkinson’s disease (OR = 0.354, 95% CI, 0.215–0.582; P <0.001), and type 2 diabetes (OR = 0.759, 95% CI, 0.637–0.905; P = 0.002). Conversely, a positive causal relationship was found between IL6Ri and asthma (OR = 1.327, 95% CI, 1.118–1.576; P = 0.001), and asthmatic pneumonia (OR = 1.823, 95% CI, 1.246–2.666; P = 0.002 (**Figure 2** and **Supplementary Material 1**: **Supplementary Table S3**).The BWMR method were consistent with those based on the IVW method (**Figure 3A** and **Supplementary Material 1**: **Supplementary Table S4**). And the effect of each SNP locus on these diseases is shown in **Figures 4**, **5**. Moreover, the robust statistical power of 100% for all these IVW results emphasizes the findings’ reliability (**Supplementary File 1**: **Supplementary Table S4**). Diseases such as ulcerative colitis, crohn’s disease, systemic lupus erythematosus, and cor pulmonale had p-values greater than 0.05 and were excluded from further analysis. Moreover, non-small cell and small cell lung cancers were excluded due to inconsistent effect directions across the six methods.

Complex pathophysiological mechanisms involving multiple factors in conditions such as cor pulmonale, non-small cell lung cancer, Alzheimer’s disease, type 1 diabetes, and inflammatory bowel disease, which includes both ulcerative colitis and Crohn’s disease, potentially obscure the efficacy of IL6Ri. In cor pulmonale, factors like chronic obstructive pulmonary disease and pulmonary embolism might interact with or independently influence the disease progression, complicating the role of IL6Ri. Non-small cell lung cancer, driven by mutations in numerous oncogenes and tumor suppressor genes, may diminish the impact of IL6Ri, overshadowed by other predominant genetic factors. The complex etiology of Alzheimer’s disease involving amyloid-beta deposition and neuroinflammation suggests that while IL6Ri may participate in controlling inflammation, its overall role in disease progression is limited. Similarly, in type 1 diabetes and inflammatory bowel disease, despite the role of IL6R in immune modulation, the effects of IL6Ri are likely moderated by other significant genetic or environmental factors, reducing its singular impact. Thus, even though IL6Ri demonstrates potential therapeutic promise, its actual effectiveness in these complex diseases may be limited by interference from other pathophysiological mechanisms.

### Effect of sIL-6R representing the IL-6 trans-signaling pathway on five diseases

Extensive literature suggests that the IL-6 trans-signaling pathway plays a pivotal role in the IL-6 signaling cascade within coronary artery disease (23–26). MR analysis, utilizing rs2228145 as the sole instrumental variable, has demonstrated that attenuated IL-6 signaling can mitigate the risk of coronary artery disease (27). The rs2228145 variant, situated on the IL6R gene, modulates the proteolytic cleavage of membrane-bound IL6R into its soluble form (sIL-6R) (28). Consequently, we selected rs2228145 as an instrumental variable for sIL-6R to investigate the impact of the IL-6 trans-signaling pathway on the disease.

The influence of sIL-6R on Type 2 diabetes (OR: 1.298, 95% CI: 1.025-1.643; P=0.030), Parkinson’s disease (OR: 3.570, 95% CI: 1.653-7.709; P= 0.001), idiopathic pulmonary fibrosis (OR: 3.308, 95% CI: 1.178-9.292; P= 0.023), asthma (OR: 0.713, 95% CI: 0.548-0.927; P= 0.011), and asthmatic pneumonia(OR: 0.438, 95% CI: 0.277-0.693; P<0.001), suggests that IL-6 receptor blockade may modulate these five diseases by inhibiting the IL-6 trans-signaling pathway(**Supplementary Material 1**: **Supplementary Table S5**).

### Sensitivity analyses

**Figure 6:**
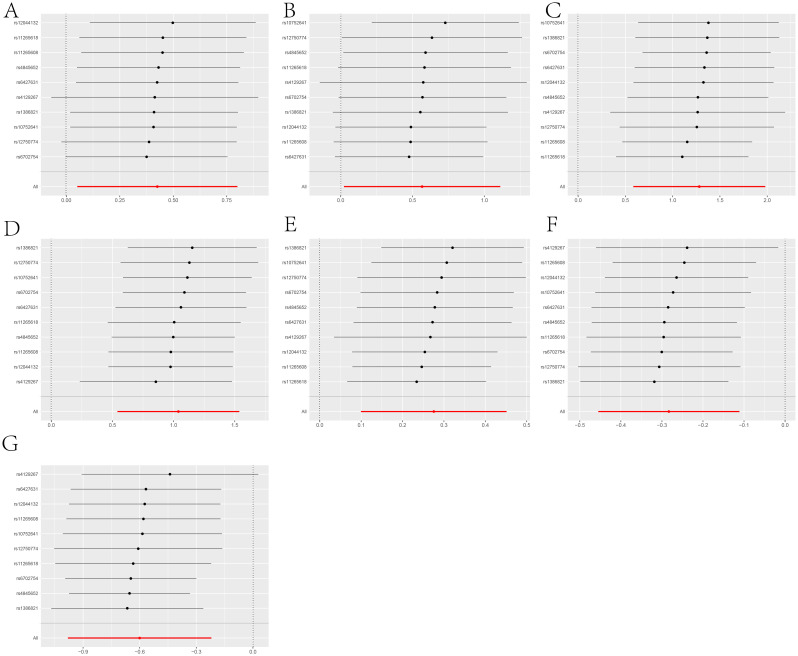
A leave-one-out analysis of statistically significant differences in the causal effects of IL-6R inhibitors on seven types of diseases, including: **(A)** COVID-19, **(B)** Rheumatoid arthritis, **(C)** Idiopathic pulmonary fibrosis, **(D)** Parkinson’s disease, **(E)** Type 2 diabetes, **(F)** Asthma, **(G)** Asthmatic pneumonia.The Y-axis corresponds to each excluded rsid number and the ‘all’ condition not excluded by the IVW method. The X-axis corresponds to specific IVW values, with black and red dots representing beta effect values, and the lines indicating the confidence intervals of these beta values.

**Figure 7:**
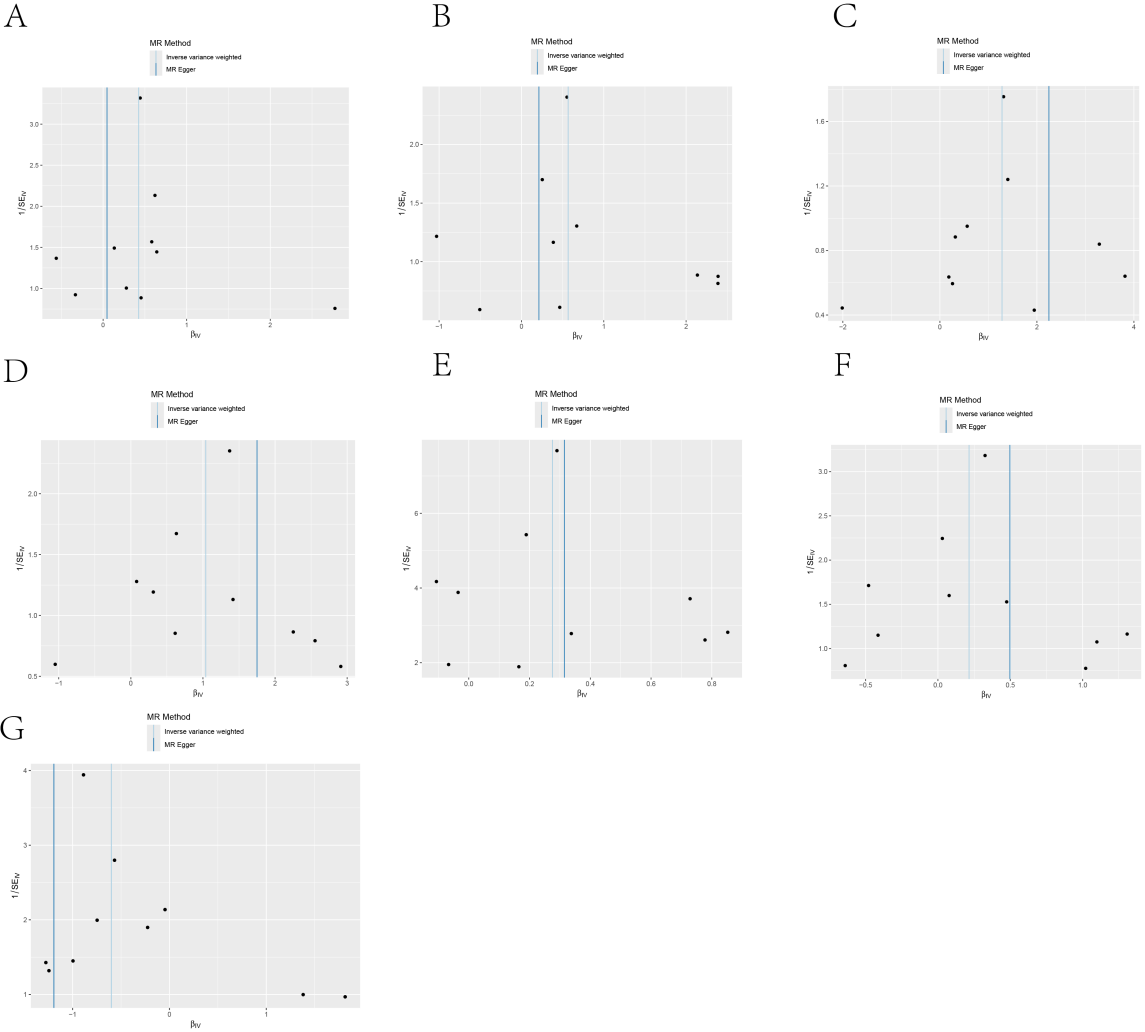
Funnel plots showing the symmetry of the causal effect of IL-6R inhibitors on seven cancers, including: **(A)** COVID-19, **(B)** Rheumatoid arthritis, **(C)** Idiopathic pulmonary fibrosis, **(D)** Parkinson’s disease, **(E)** Type 2 diabetes, **(F)** Asthma, **(G)** Asthmatic pneumonia.The black dots in the figure represent single nucleotide polymorphisms, with the horizontal axis displaying the β values of these polymorphisms, and the vertical axis showing their standard errors.

Leave-one-out analysis demonstrated consistent risk estimates for idiopathic pulmonary fibrosis, Parkinson’s disease, type 2 diabetes, asthma, and asthmatic pneumonia after sequentially removing each SNP (**Figure 6**), and the funnel plots appeared approximately symmetrical(**Figure 7**).Cochran’s Q test did not indicate any signs of heterogeneity. Furthermore, neither MR-Egger regression nor MR-PRESSO detected the presence of horizontal pleiotropy (**Figure 2** and **Supplementary Material 1**: **Supplementary Table S3**).We further investigated the colocalization of idiopathic pulmonary fibrosis, asthma, asthmatic pneumonia, Parkinson’s disease, and type 2 diabetes with CRP. The colocalization evidence for asthmatic pneumonia (coloc.abf-PPH4 = 0.811) strongly supports its association with CRP. For Parkinson’s disease, the colocalization evidence (coloc.abf-PPH4 = 0.725) moderately supports a link with CRP. However, the evidence for idiopathic pulmonary fibrosis (coloc.abf-PPH4 = 0.222), asthma (coloc.abf-PPH4 = 0.123), and type 2 diabetes (coloc.abf-PPH4 = 0.062) shows limited colocalization (**Figure 3B** and **Supplementary Material 1**: **Supplementary Table S6**).

Positive results from the colocalization analysis have revealed shared genetic signals within the genetic locus linking CRP with Parkinson’s disease and asthmatic pneumonia. These genetic signals are located in the IL6R gene region. These findings suggest that genetic variations in the IL6R region may influence the development of Parkinson’s disease and asthmatic pneumonia by modulating inflammation-related biological pathways.

## Discussion

In this study, we conducted a systematic analysis of the causal relationships between IL-6Ri and various diseases. To our knowledge, this represents the first large-scale genetic consortium-based MR analysis to establish causal links between IL-6Ri and multiple diseases. Utilizing drug-target Mendelian Randomization and colocalization analyses, we concluded that IL-6Ri significantly reduce the incidence risk of Parkinson’s disease, idiopathic pulmonary fibrosis, and type 2 diabetes, while potentially increasing the risk of asthma and asthmatic pneumonia.

Parkinson’s disease is the most common severe motor disorder and the second most prevalent neurodegenerative disease after Alzheimer’s, affecting 400 to 1,900 individuals per 100,000 globally (29). With the aging population, the incidence of Parkinson’s disease is expected to double by 2040 (30). In research conducted by Chen et al. (31), an analysis of 84 Parkinson’s patients and 165 control samples revealed that plasma inflammation markers—including C-reactive protein, fibrinogen, tumor necrosis factor-alpha, and IL-6—are critical for predicting and diagnosing Parkinson’s disease, particularly as elevated IL-6 significantly increases the risk of developing the disease. Studies indicate that IL-6 activates the JAK-STAT pathway by increasing pSTAT3 expression (32). QingQin et al. confirmed that overexpression of α-SYN in a Parkinson’s disease model activates the JAK/STAT pathway. Inhibition of the JAK/STAT pathway, demonstrated for the first time, disrupts the circuits of neuroinflammation and neurodegenerative changes, thereby mitigating the pathogenesis of Parkinson’s disease (33). In a Parkinson’s disease rat model, inhibitors of JAK1 and JAK2, such as AZD1480, reduced microglial proliferation and macrophage infiltration and decreased MHC class II expression. Additionally, treatment with AZD1480 inhibited the activation of STAT1/3/4 and blocked the differentiation of Th1 and Th17 cells, jointly promoting an immune response in the Parkinson’s disease model. In this study, we are excited to report that IL6Ri can reduce the risk of Parkinson’s disease, and colocalization showed moderate strength. The results of colocalization analysis further confirm that the role of IL6R in the nervous system may be related to its anti-inflammatory properties, providing significant insights into the specific mechanisms of IL6R in disease. Our research underscores the potential value of targeting the IL6R pathway in the prevention and treatment of Parkinson’s disease.

Idiopathic Pulmonary Fibrosis is a chronic fibrosing interstitial pneumonia of unknown origin that predominantly affects the elderly, characterized by progressive respiratory difficulty and continual decline in lung function. Prognosis for patients is poor, with an average life expectancy of about three to five years post-diagnosis (34). According to the Global Burden of Disease study, as of 2019, the incidence rate of interstitial lung diseases and pulmonary sarcoidosis among Chinese men was approximately 65 cases per 100,000 people, while globally, the incidence rates for men and women were 68 and 59 per 100,000 respectively. With the increasing trend of an aging population, the number of individuals with Idiopathic Pulmonary Fibrosis in China is expected to reach at least 500,000. In developed regions like the European Union, the number of Idiopathic Pulmonary Fibrosis patients increases by about 3,500 annually, posing a significant socioeconomic burden and an urgent health care challenge worldwide (35). The JAK/STAT signaling pathway is one of the classic inflammatory pathways closely associated with the development of Idiopathic Pulmonary Fibrosis (36). In this pathway, when the JAK1/STAT1 pathway is activated, JAK1 becomes phosphorylated, which in turn induces the phosphorylation of STAT1. The phosphorylated STAT1 forms heterodimers and translocates to the nucleus, promoting the release of chemokines and pro-inflammatory cytokines such as TNF-α, IL-6, ICAM1, and MCP1 (37). These inflammatory mediators have been identified as potential therapeutic targets for various fibrotic diseases. Furthermore, the SOCS family proteins serve as important negative regulators within the JAK1/STAT1 pathway. As negative regulators of cytokine receptor signaling, they inhibit the overactivation of the JAK/STAT pathway. This feedback inhibition mechanism plays a crucial role in regulating inflammatory responses and preventing excessive tissue fibrosis (38). Therefore, the potential of IL6Ri to reduce the risk of developing Idiopathic Pulmonary Fibrosis may be attributed to their ability to inhibit the JAK-STAT pathway, though further *in vivo* and *in vitro* studies are necessary to validate this effect.

Diabetes is an endocrine and metabolic disorder characterized by defects in insulin secretion and/or action and chronic hyperglycemia, caused by various factors (39). Epidemiological studies indicate that approximately 537 million people worldwide suffer from diabetes, with projections suggesting this number will rise to 693 million by 2045 (40). The global economic burden of adult diabetes is substantial, with costs estimated at 1.3 trillion USD in 2015, expected to increase to between 2.1 and 2.8 trillion USD by 2030 (41). Since 1999, research has progressively revealed a close association between type 2 diabetes mellitus and inflammation, recognizing the disease as an inflammatory condition mediated by inflammatory cells, their secretory factors, and acute-phase reactants, constituting an innate immune response. Particularly, chronic inflammation of visceral adipose tissue plays a pivotal role in the pathogenesis of type 2 diabetes. Obesity triggers adipocyte hypertrophy and hyperplasia, reduces the anti-inflammatory factor APN, and increases pro-inflammatory cytokines such as IL-6, IL-1β, TNF-α, and chemokines (CCL2, CCL3, and CXCL8), leading to the infiltration of immune cells (M1-type macrophages, CD8+ and CD4+ T lymphocytes, and B lymphocytes) and consequently promoting adipose inflammation (42). Our studies suggest that IL-6 inhibitors can significantly reduce type 2 diabetes, potentially through the inhibition of the JAK-STAT signaling pathway, thereby alleviating insulin resistance.

Common side effects of IL-6Ri include nasopharyngitis, headaches, upper respiratory tract infections, and gastritis. Infections are the most frequently occurring serious side effect, which may lead to gastrointestinal perforations. Typical laboratory abnormalities include neutropenia and elevated liver enzymes (43). Local reactions to injections and infusion reactions are common, but systemic allergic reactions are rare (44, 45). In rheumatoid arthritis patients, the development of resistance antibodies is possible, though they do not affect the efficacy of the treatment. Clinical trials indicate that IL-6Ri increase the risk of infections, consistent with other similar medications, including serious infections such as bacterial pneumonia and atypical infections (46). Interestingly, our research suggests a potential increase in the risk of asthma and asthmatic pneumonia, providing crucial information for clinicians in making therapeutic decisions, especially when considering pre-existing respiratory conditions in patients. Therefore, it is recommended that patients using these inhibitors be closely monitored for potential respiratory complications.

Currently available IL6R inhibitors, such as Tocilizumab and Sarilumab, operate by targeting the IL-6 receptor, effectively obstructing the interaction between IL-6 and its receptor, thus inhibiting both the classical and trans-signaling pathways. These inhibitors not only reduce the availability of membrane-bound IL-6R but also decrease the activity post-binding to sIL-6R, showcasing their suppressive effects across both signaling pathways (28). Conceptually, our genetic tools mirror the actions of these anti-IL6R monoclonal antibodies, although they exhibit a comparatively weaker effect in inhibiting IL6R signaling. Nevertheless, they possess a profound conceptual similarity with IL-6 inhibition. Empirical data supports the variation at this site and its impact on subsequent randomized trial data. Particularly when we employ the “canonical” rs2228145 SNP for MR analysis, despite reduced experimental efficacy, the outcomes align with expectations, exhibiting similar effects. Moreover, Tocilizumab, based on the IgG1 subtype, activates a variety of immune responses, such as antibody-dependent cell-mediated cytotoxicity and complement-dependent cytotoxicity. In contrast, Sarilumab, derived from the IgG2 subtype, exhibits lower activity in activating these immune effects. Both Tocilizumab and Sarilumab demonstrate similar therapeutic effects (47); however, Sarilumab presents a reduced risk of side effects (48). Therefore, we remain optimistic about the potential of these inhibitors in treating conditions such as idiopathic pulmonary fibrosis, type 2 diabetes, and Parkinson’s disease, anticipating that Sarilumab’s side effects, particularly in asthma and asthmatic pneumonia, may be significantly lesser than those of Tocilizumab.

The primary limitations of this study stem from the fact that the causal relationships are based on “genetic predispositions,” which can be easily influenced if the instrumental variable data are not properly managed. Due to constraints related to databases and software, this study focused exclusively on Europeans, but the causal conclusions drawn from European populations may not necessarily apply to other groups, such as Asians. Future studies should verify these findings using larger samples across multiple regions and diverse ethnic populations.

## Conclusion

In summary, this study assessed the causal relationships between IL-6Ri and various diseases, discovering that IL-6Ri can reduce the risk of three diseases while potentially increasing the risk of asthma and asthmatic pneumonia. This provides preliminary evidence for the potential expansion of therapeutic applications for IL-6Ri.

## Data Availability

The original contributions presented in the study are included in the article/**Supplementary Material**. Further inquiries can be directed to the corresponding author.
